# Substance P enhances the therapeutic effect of MSCs by modulating their angiogenic potential

**DOI:** 10.1111/jcmm.15804

**Published:** 2020-09-28

**Authors:** Hyun Sook Hong, Suna Kim, Yinji Jin, Youngsook Son

**Affiliations:** ^1^ Department of Biomedical Science and Technology Graduate School Kyung Hee University Seoul Korea; ^2^ East‐West Medical Research Institute Kyung Hee University Hospital Seoul Korea; ^3^ Kyung Hee Institute of Regenerative Medicine (KIRM) Medical Science Research Institute Kyung Hee University Medical Center Seoul Korea; ^4^ Department of Genetic Engineering College of Life Science Graduate School of Biotechnology Kyung Hee University Yong In Korea

**Keywords:** mesenchymal stem cell, paracrine factors, pericytes, substance P

## Abstract

Bone marrow mesenchymal stem cell (MSC) therapy acts through multiple differentiations in damaged tissue or via secretion of paracrine factors, as demonstrated in various inflammatory and ischaemic diseases. However, long‐term ex vivo culture to obtain a sufficient number of cells in MSC transplantation leads to cellular senescence, deficiency of the paracrine potential, and loss of survival rate post‐transplantation. In this study, we evaluated whether supplementation of MSCs with substance P (SP) can improve their therapeutic potential. SP treatment elevated the secretion of paracrine/angiogenic factors, including VEGF, SDF‐1a and PDGF‐BB, from late passage MSCs in vitro. MSCs supplemented with SP accelerated epidermal/dermal regeneration and neovascularization and suppressed inflammation in vivo, compared to MSCs transplanted alone. Importantly, supplementation with SP enabled the incorporation of transplanted human MSCs into the host vasculature as pericytes via PDGF signalling, leading to the direct engagement of transplanted cells in compact vasculature formation. Our results showed that SP is capable of restoring the cellular potential of senescent stem cells, possibly by modulating the generation of paracrine factors from MSCs, which might accelerate MSC‐mediated tissue repair. Thus, SP is anticipated to be a potential beneficial agent in MSC therapy for inflammatory or ischaemic diseases and cutaneous wounds.

## 1 INTRODUCTION

Transplantation of mesenchymal stem cells (MSCs), also known as multipotent stromal cells, is performed to repair intractable tissue in the bone, skin, eye, heart and kidney.^1^, ^2^, ^3^, ^4^, ^5^, ^6^, ^7^, ^8^, ^9^ MSCs can affect different components of the immune system to ameliorate severe inflammatory responses, presumably by secreting various paracrine factors, including interleukin‐10, transforming growth factor β1 (TGF‐β1), indoleamine 2, 3‐dioxygenase and prostaglandin E2.^10^, ^11^, ^12^, ^13^, ^14^, ^15^, ^16^, ^17^ In addition to their immunosuppressive role, MSCs share markers specific to pericytes and act as pericytes,^18^ indicating the direct involvement of MSCs in vascularization. Accordingly, the immune modulation and angiogenic potential of MSCs has been regarded as the main operating mechanisms of MSC therapy.

In spite of the mounting evidence for the therapeutic effects of MSCs, the low differentiation potential to damaged tissue and transient persistence of transplanted MSCs have emerged as crucial problems that need to be addressed. A sufficient number of MSCs are required for transplantation, which in turn requires long‐term ex vivo culture and makes transplantation of late‐passage MSCs inevitable. During ex vivo culture, MSCs become senescent, with aberrant biological characteristics such as morphological changes, reduced proliferation rate, deficient immunosuppressive function and insufficient production of cytokines/growth factors such as stromal cell‐derived factor‐1 alpha (SDF‐1α) or vascular endothelial growth factor (VEGF). ^19^, ^20^, ^21^, ^22^, ^23^ Moreover, because senescent MSCs encounter severe inflammatory environments in vivo post‐transplantation, they do not survive long term. Therefore, transplanted MSCs have impaired therapeutic function. To address these problems, the current research focuses on ways to enhance viability and inhibit senescence of MSCs using specific growth factors, co‐culture with supportive cells, hypoxic conditions and genetic modification.^22^, ^23^, ^24^, ^25^ However, effective methods to improve the efficacy of stem cell therapy in vivo have not been optimized.

Substance P (SP), an endogenous neuropeptide known to be involved in neuro‐immune modulation, can also promote cell proliferation and inhibit cell apoptosis.^26^, ^27^, ^28^ Hong et al demonstrated that SP can induce mobilization of MSCs into the circulation by repopulating MSCs in the bone marrow, leading to tissue repair.^29^ SP promotes anti‐inflammatory responses that ameliorate disease progression in cases of corneal wounds, rheumatoid arthritis, radiation‐induced intestinal damage and spinal cord injury.^29^, ^30^, ^31^, ^32^, ^33^ When MSCs are treated with SP in vitro, the production of VEGF and fibronectin in MSCs increases.^29^ Recent studies have demonstrated that SP can restore the immunosuppressive function of late‐passage MSCs by blocking senescence‐induced reduction of cytokine secretion from MSCs.^23^

Our approach was based on the hypothesis that the addition of SP to transplanted MSCs adequately enhances the efficacy of MSC therapeutics in vivo, possibly by modulating the secretion of paracrine factors and enhancing the survival of MSCs at the injured site.

To determine the effect of SP on the paracrine potential of senescent MSCs, late‐passage human MSCs were treated with SP and changes in neovascularization‐related growth factors were evaluated by ELISA in vitro. To elucidate the beneficial effect of SP on MSC‐mediated neovascularization and wound repair in vivo, late‐passage MSCs were transplanted with SP into full‐thickness cutaneous wounds, and wound healing was assessed by analysing epidermal and dermal recovery, collagen deposits, immune cell infiltration, neovascularization and systemic inflammatory responses, in comparison to wounds treated with vehicle or MSC transplantation without SP.

## 2 MATERIALS AND METHODS

### 2.1 Materials

MSCGM was purchased from Lonza (Basel, Switzerland); phosphate‐buffered saline, (PBS), Welgene (Daegu, Korea); SP, Sigma‐Aldrich (St. Louis, MO, USA). ELISA kits for SDF‐1α, platelet‐derived growth factor‐BB (PDGF‐BB), and VEGF were obtained from R&D systems (Minneapolis, MN, USA). Tegaderm Film was purchased from 3M Health Care (St. Paul, MN, USA) and Mepitel, from Molnlycke Health Care (Gothenburg, Sweden).

### 2.2 Experimental animals

Five‐week‐old nude mice were obtained from DBL (Daehan Bio Link, Seoul, Korea). Animals were maintained under a 12‐h light/dark illumination cycle in the animal facility and allowed to acclimatize to the new environment for 1 week. All animal studies were approved by the Ethical Committees for Experimental Animals at Kyung Hee University (KHMC‐IACUC‐14‐010).

### 2.3 MSC culture

A bone marrow sample was obtained from healthy human donor (age: 45‐65 years, 6 donors. 3 females, 3 males). Human bone marrow aspirates (BMAs, 10 mL) were obtained with the written consent of patients and the protocol was approved by the St. Peter's Hospital Institutional Review Board (IRB#: KPH 2010‐01).

Primary MSCs were harvested by separating mononuclear cells from the bone marrow sample via density‐gradient centrifugation: Human bone marrow specimens were diluted in PBS, overlaid on Ficoll‐Paque solution (GE Healthcare, Buckinghamshire, UK) and centrifuged at 2200 rpm for 20 min. The mononuclear cell layer at the interface was harvested and washed twice with PBS. Cells were suspended in MSC growth medium (MSCGM) and cultured under 5% _CO2_ at 37°C. After 48 h, erythrocytes and non‐adherent cells were eliminated via a PBS wash. On approaching 70% confluence, cells were detached with 0.25% trypsin/ EDTA (Welgene, Daegu, Korea) and sub‐cultured at 2.5 × 10^5^ cells per 100‐mm dish.

### 2.4 Cytokine measurements

Secretion of SDF‐1α, PDGF‐BB and VEGF from MSCs was quantified using ELISA kits in accordance with the manufacturer's instructions. In brief, MSCs were seeded in 24‐well plates at 2 × 10^4^ cells/well and treated with SP (100 nM) or PBS (control) for 48 h. Conditioned media were harvested and centrifuged to eliminate cell debris. To quantify cytokine levels, conditioned media and standards were added to 96‐well plates coated with anti‐SDF‐1α, anti‐PDGF‐BB and anti‐VEGF antibodies, followed by incubation for 2 h. After four washes with PBS, horseradish peroxidase‐conjugated secondary antibodies were added to each well for 2 h at room temperature. After three more washes, the substrate solution was added and the reaction was allowed to proceed for 30 min; thereafter, the reaction was terminated using stop solution. Absorbance was measured at 450 nm with an EMax Endpoint Microplate Reader (Molecular Devices, Sunnyvale, CA, USA).

### 2.5 Establishment of a mouse model of a full‐thickness skin wound

Nude mice (25 g, male, BALB/c nude mice, N = 8 for each group) were randomly divided into 4 groups: Untreated, vehicle‐treated, MSC (p8)‐treated and MSC (p8) + SP‐treated groups. A dorsal 8‐mm‐thick wound was inflicted under anaesthesia with ketamine (100 mg/kg, Yuhan co., Korea) and rumpun (1.2 mg/kg, Bayer Healthcare, Korea). The wound was covered with Mepitel and Tegaderm.

### 2.6 Cell transplantation

At day 3 post‐wound infliction, 5 × 10^5^ MSCs in hydrogel (Poloxamer 407, BASF, Lutrol F 127, volume 0.04 ml, 2.5 g/ml) with or without SP (final, 100 nM) were transplanted topically, using hydrogel as the vehicle. AG1295 (2 μM, PDGF tyrosine receptor kinase inhibitor, Santa Cruz, Dallas, TX, USA) was co‐treated with SP to inhibit PDGF‐PDGFR signalling in transplanted MSCs. The safety of AG1295 was then assessed (Figure S1).

### 2.7 Haematoxylin & Eosin (H&E) staining

At days 2 and 7 post‐transplantation, mice were euthanized and the skin and spleen were harvested for further quantitative and histological analysis. The skin and spleen were fixed in 3.7% formaldehyde for 24 h and embedded in paraffin, and the paraffin‐embedded tissue blocks were processed with a TP1020 tissue processor (Leica Biosystems, Wetzlar, Germany). For histological analysis, 4‐μm‐thick sections were deparaffinized in xylene and rehydrated using an alcohol gradient. For H&E staining, nuclei were stained with haematoxylin (Sigma‐Aldrich, St. Louis, MO, USA) for 1 min, after which the sections were washed in running water for 5 min, and then, the cytoplasm and extracellular matrix were stained with eosin Y (Sigma‐Aldrich) for 10 s.

### 2.8 Masson trichrome (MTC) staining

To observe collagen deposition in the wound region, MTC Staining (IHC World, Woodstock, MD, USA) was performed in accordance with the manufacturer's instructions. In brief, paraffinized tissue sections (4‐μm thick) were deparaffinized, hydrated and fixed in Bouin's solution (Sigma‐Aldrich) for 1 h at 56°C. After washing in distilled water, the samples were stained with Weigert's haematoxylin for 10 min, Biebrich scarlet‐acid fuchsin for 2 min, phosphomolybdic‐phosphotungstic acid solution for 10 min, and Aniline Blue for 10 min. After washing with distilled water, samples were treated with 1% acetic acid (Sigma‐Aldrich) for 5 min and then dehydrated and observed using a microscope.

### 2.9 Immunohistochemical staining

Immunohistochemical staining was carried out using the VECTASTAIN ABC Kit (Vector Laboratories, Burlingame, CA, USA) in accordance with the manufacturer's instructions. Samples were treated with 0.5% H_2_O_2_ to block endogenous peroxidase activity for 10 min and permeabilized with 0.3% Triton‐X100. Non‐specific binding was inhibited by incubating samples with 1% normal horse serum for 1 h at room temperature (RT). Primary mouse anti‐CD31, anti‐F4/80 and anti‐SDF‐1α antibodies (Abcam, Cambridge, MA, USA) were added. After three PBS washes, samples were incubated with a biotin‐conjugated secondary antibody for 1 h at RT. After washing with PBS, the substrate solution was added and the reaction was allowed to proceed at RT for 40 min. To visualize the reactive area in the tissue, samples were treated with dimethyl‐aminoazobenzene (DAB; Vector Laboratories), counterstained with Fast Red (Vector Laboratories) for 10 min and mounted. Finally, samples were observed using a Nuance Multiplex Biomarker Imaging System (Cambridge Research Instrumentation, Woburn, MA, USA).

### 2.10 Immunofluorescence staining

Wounded tissues were embedded in optical cutting compound (OCT compound, Sakura Finetek, Tokyo, Japan) and cut into 4‐μm‐thick sections. To eliminate OCT compound, sections were washed thrice with PBS and incubated in 20% normal goat serum to block non‐specific binding. Sections were probed with anti‐mouse CD31 antibody (Abcam) for 2 h at RT, followed by FITC‐conjugated secondary antibody for 1 h. After two washes, anti‐human α‐SMA antibody (Dako, DK) was added, followed by Texas Red‐conjugated secondary antibody (Vector Laboratories). Thereafter, samples were mounted with Vectashield mounting medium (Vector Laboratories with DAPI) and observed using a Nuance Multiplex Biomarker Imaging System (Cambridge Research Instrumentation).

### 2.11 Quantitative histological analysis of the wounded area

For quantitative histologic analysis, the wounded area was assessed using previously described methods, with certain modifications^16^ Cell migration in the epithelial layer was analysed by measuring the path length of epithelial cells from the non‐wounded edge to the centre of the wound. Granulation tissue formation was evaluated on the basis of the invasion of endothelial cells, fibroblast influx, collagen deposition and additional macrophage accumulation. The thickness of the granulation tissue was measured with respect to the underlying muscle fascia. Wound repair is expressed as an absolute value. All quantitative analyses were performed using ten adjacent fields on temporal slides.

### 2.12 Statistical analysis

All data are presented as the mean ± standard deviation (SD). Student's t test (for comparisons between two groups) and one‐way analysis of variance (ANOVA; for comparisons of three or more groups, followed by Tukey's post‐hoc test) were performed. Probability values less than 0.05 were interpreted to indicate statistically significant differences (*P < .05, **P < .01, ***P < .001).

## 3 RESULTS

### 3.1 SP stimulates the secretion of paracrine/ angiogenic factors in late‐passage MSC*s*

Paracrine factors from MSCs, rather than the MSCs themselves, are chiefly responsible for tissue repair. SDF‐1α is the primary chemokine that supports mobilization of pro‐angiogenic cells, including endothelial progenitor cells, and is capable of accelerating vascularization.^34^ VEGF, a mitogen highly specific for vascular endothelial cells, induces proliferation, promotes migration and inhibits apoptosis of endothelial cells. MSCs are known to secrete SDF‐1α and VEGF constitutively, but the secretion of these paracrine factors reduces as the passage number increases; this might be the main cause of the low efficacy of MSC therapeutics until now.^14^

Before SP treatment, activity of BM MSC was evaluated according to passage number. BM MSCs at early (p3) and late passage (p8) were characterized by FACS analysis and then treated with SP. FACS analysis indicated no differences in cell surface marker expression between the early‐ and late‐passage MSCs (Table S1). Cellular senescence was determined by beta galactosidase staining. Even though its portion was not as high, beta galactosidase + cells appeared from passage 5 BM MSC and it was undeniable at passage 8 BM MSC. That is, passage 8 BM MSC might undergo senescent states (Figure S2). Moreover, cellular activity was reduced and doubling time was firmly elevated at passage 8 BM MSC. Moreover, immune suppressive function was clearly impaired at passage 8 BM MSC, comparing to passage 3 BM MSC (Figure S3 and Figure S4).

To determine whether SP can affect the secretion of paracrine/angiogenic factors from late‐passage MSCs, passage 8 BM MSC was treated with SP in this study.

**Figure 1:**
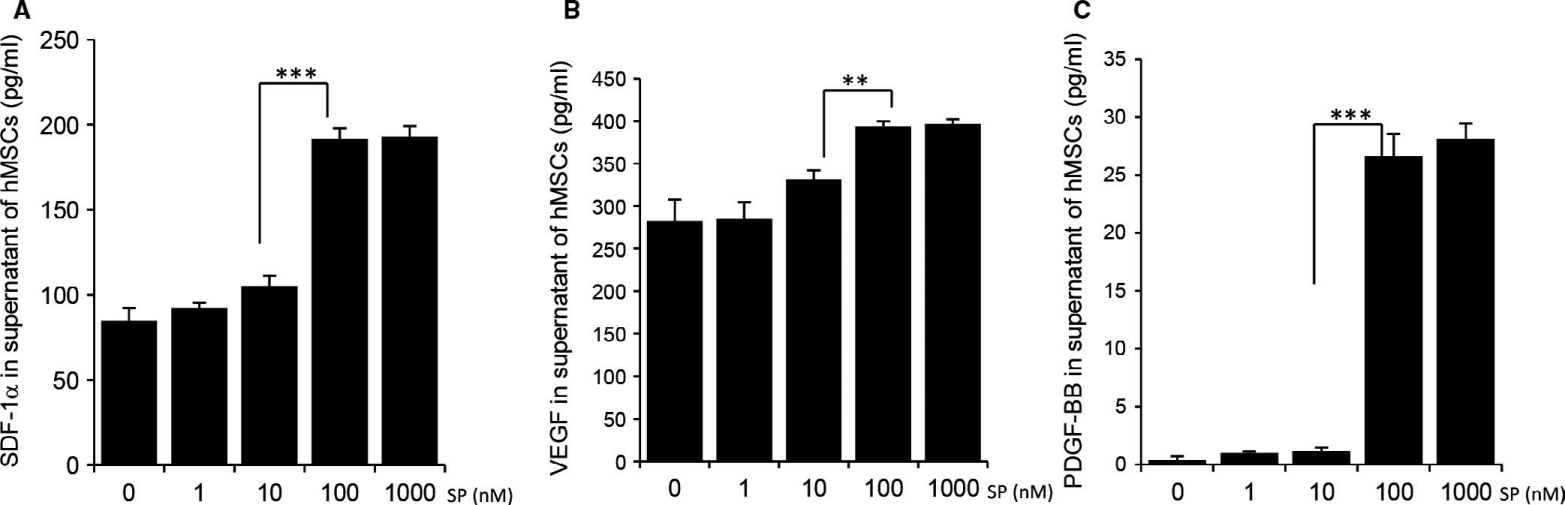
SP promotes secretion of angiogenic factors in late passage MSCs in vitro. MSCs at passages 8 were inoculated in 24‐well plates at a concentration of 2 × 10^4^ cells/well. SP (1, 10, 100 and 1000 nM) was added to p8 MSCs and 24 h later, conditioned medium was collected. The amounts of SDF‐1α (A), VEGF (B) and PDGF‐BB (C) in the MSC‐conditioned medium were measured using ELISA. Values of *P* < .05 were considered statistically significant (*P < .05, **P < .01, ***P < .001). The data are represented as mean ± SD of three independent experiments

Levels of SDF‐1α and VEGF in the culture supernatant were determined by ELISA. As the passage number increased, the release of SDF‐1α and VEGF by MSCs decreased (Figure S5). SP treatment increased the secretion of SDF‐1α and VEGF in late‐passage MSCs to levels resembling early‐passage MSCs (Figure 1A and B). SP effect was dose‐dependent.

Pericytes regulate vasoconstriction and vasodilation within capillary beds to control vascular diameter and capillary blood flow.^18^, ^35^, ^36^ Dysfunctional and leaky blood vessels that lack pericytes contribute to the development of pathological conditions in vivo. PDGF‐BB is involved in the recruitment of pericytes to a variety of vascular beds such as the brain, kidney, heart, lung and adipose tissue, ^35^, ^37^, ^38^ leading to the formation of a healthy and compact vasculature encircled with pericytes. Moreover, PDGF‐BB is found to protect MSCs against apoptosis and senescence. Inhibition of platelet‐derived growth factor receptor suppressed the proliferation and altered the differentiation of MSCs.^39^, ^40^ Both of early‐ and late‐passage MSCs secreted PDGF‐BB at undetectable levels (data not shown). With the addition of SP to late‐passage MSCs, the secretion of PDGF‐BB was elevated (Figure 1C).

These data suggest that cell surface markers are not good surrogates to represent the cellular state. The secretion of paracrine factors from MSCs was significantly lower during ex vivo term culture, but SP treatment could restore the ability to produce paracrine factors, including SDF‐1α, VEGF and PDGF‐BB, in late‐passage MSCs in dose‐dependent manner, corresponding with neovascularization through migration of vascular endothelial cells and the incorporation of pericytes. In addition, because VEGF and PDGF‐BB are known to increase the survival of transplanted MSCs, the combination of MSCs and SP was expected to augment the therapeutic effect of transplanted MSCs by extending MSC survival. Based on Figure 1, we have selected 100nM as a dose of SP for the further experiment.

### 3.2 SP can improve MSC‐induced cutaneous wound healing

**Figure 2:**
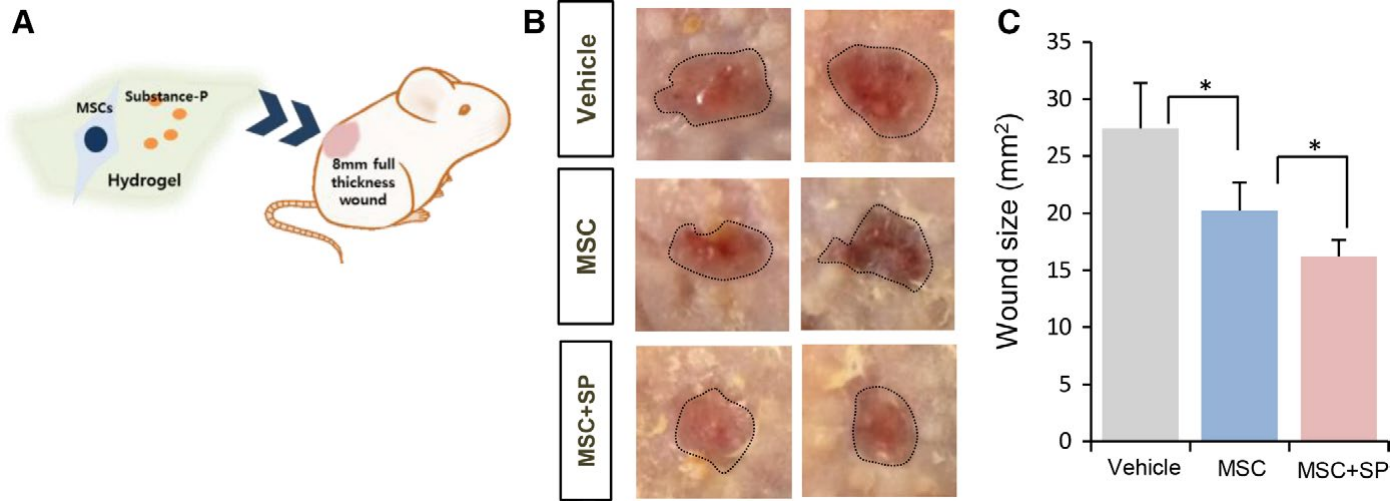
SP enhances MSC‐mediated wound closure. (A) Experimental scheme for cell transplantation. (B) Gross views of wound areas on day 7 post‐MSC transplantation with or without SP. Black dotted lines indicate wound area (C) Quantification of wound areas. Values of *P* < .05 were considered statistically significant (*P < .05, **P < .01, ***P < .001). The data are represented as mean ± SD of three independent experiments. N = 8 for each group

SP has been shown to accelerate proliferation and restore the immune modulatory function of MSCs in vitro.^23^ As shown in Figure 1, SP promoted the production of paracrine/ angiogenic factors by MSCs. To examine the beneficial role of SP in MSC therapy, cutaneous wounds of full thickness were generated, followed by transplantation with late passage MSCs with or without SP at day 3 post‐wound, when severe inflammatory responses develop (Figure 2A). Wound closure was monitored for 7 days post‐transplantation (10 days post‐wounds). Photographs were taken to document the time‐dependent reduction in wound size (Figure 2B). MSC transplantation accelerated wound closure compared with vehicle‐treated wounds, and the addition of SP to MSCs promoted even faster wound closure (Figure 2C; day 7 post‐transplantation, vehicle: 27.4 ± 4 mm^2^, MSC: 20.2 ± 2.4mm^2^, MSC + SP: 16.2 ± 1.4 mm^2^, MSC vs. MSC + SP *P* < .05), supporting the effect of SP in facilitating MSC‐induced wound closure.

**Figure 3:**
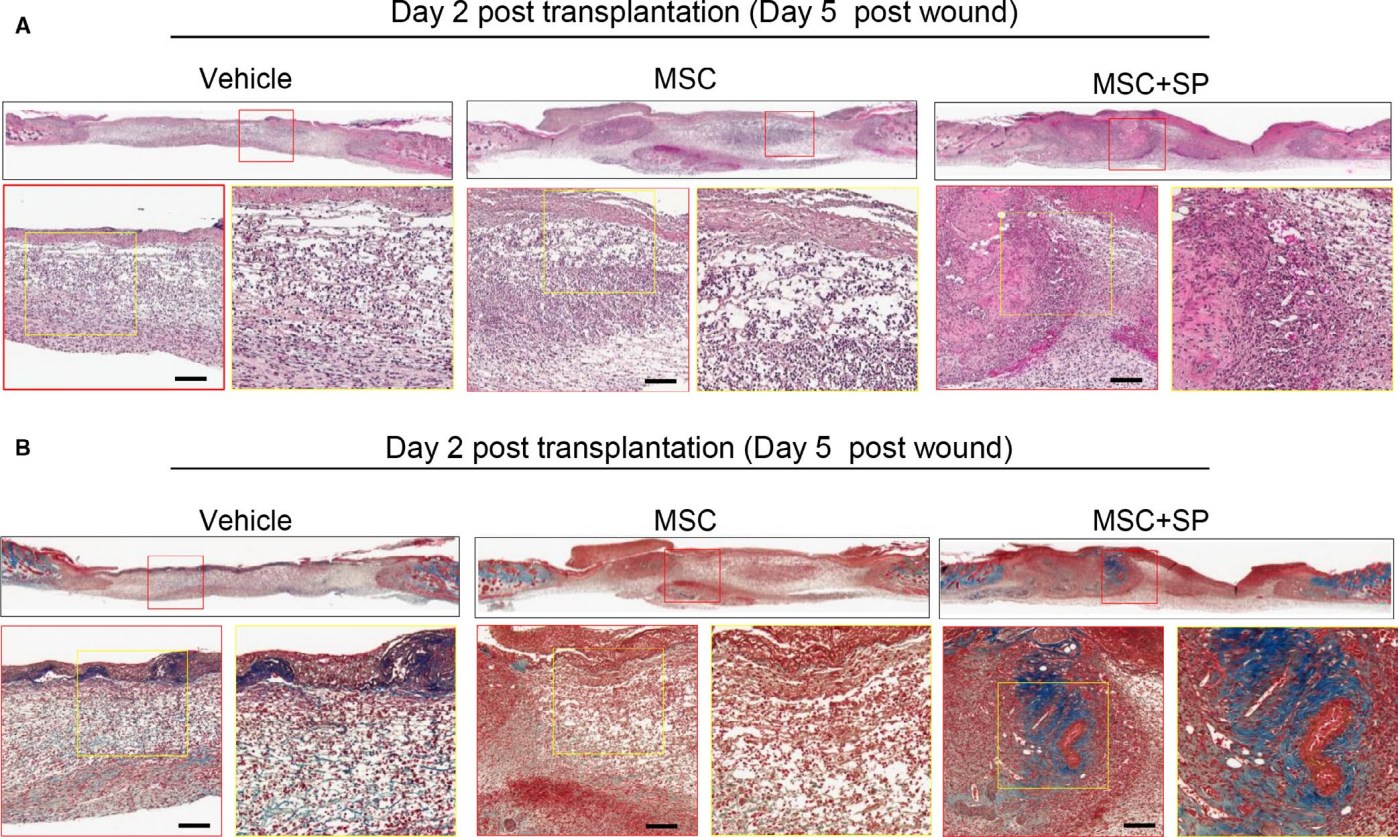
SP accelerates the regeneration of wound tissue by hastening MSC‐induced recovery of the epidermis and dermis at 2 days post‐transplantation. Histological analysis of wounded tissue was performed. (A) Samples of 4‐μm thickness were stained with haematoxylin and eosin. (B) Collagen deposition was detected by Masson trichrome staining. N = 8 for each group. Scale bar: 100 μm

**Figure 4:**
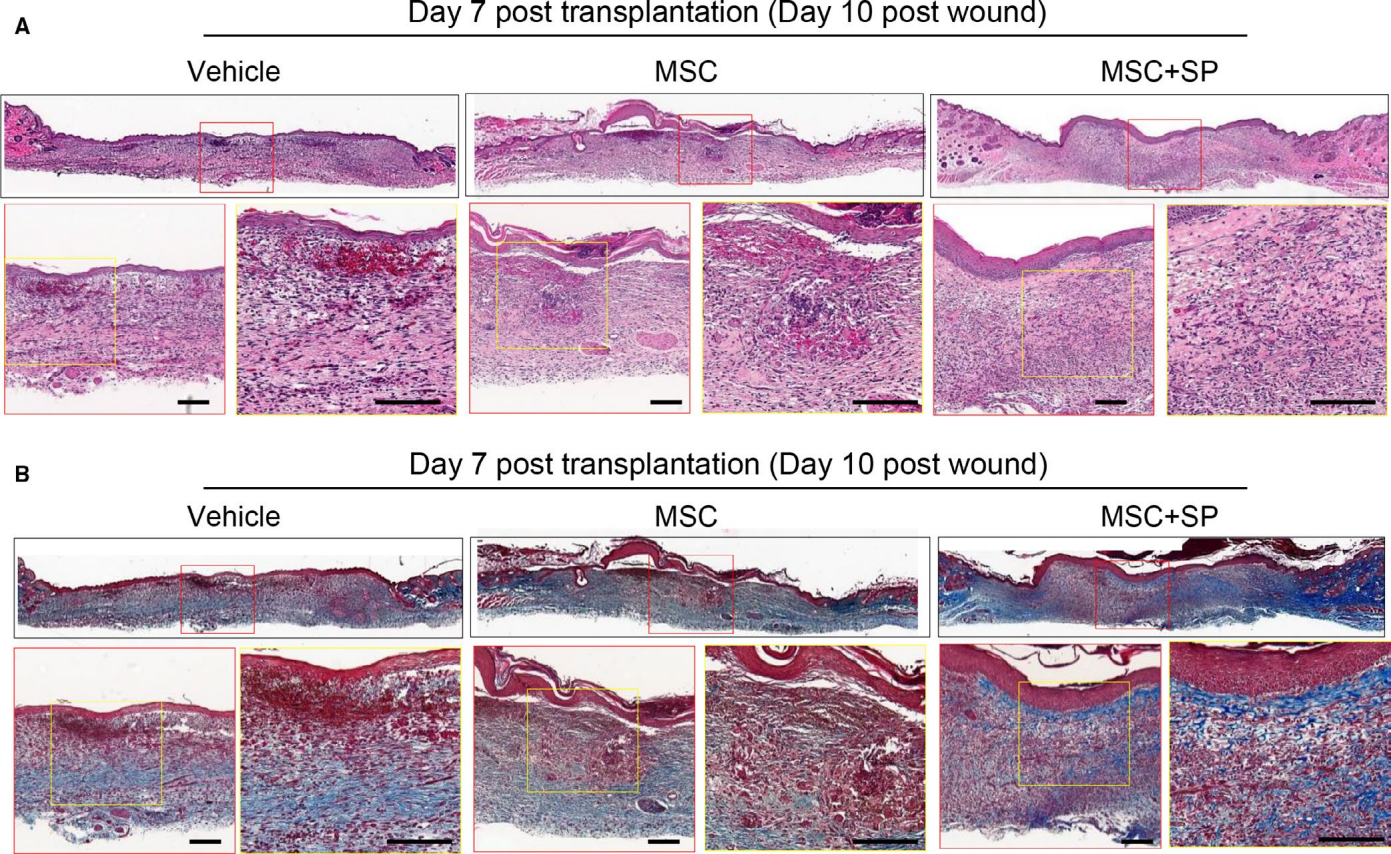
SP accelerates regeneration of wound tissue by hastening MSC‐induced recovery of the epidermis and dermis at 7 days post‐transplantation. Histological analysis of wounded tissue at day 7 post‐wound was performed. (A) Samples of 4‐μm thickness were stained with haematoxylin and eosin. (B) Collagen deposition was detected by Masson trichrome staining. Scale bar: 100 μm. N = 8 for each group. Scale bar: 100 μm

Next, wound healing was evaluated based on the histology of the injured site. At day 2 post‐transplantation, vehicle‐treated mice showed poor coverage of the epidermal layer and a sparse dermis structure, while MSC‐transplanted wounds were partially covered with epidermis with scant recovery of the dermis. On day 2, differences between the vehicle and MSC‐transplanted wounds were barely detected. Meanwhile, the combination of MSCs and SP facilitated epidermal migration and dermal regeneration with increased collagen deposition (Figure 3). On day 7 post‐transplantation, vehicle‐treated wounds had a thin epidermal layer and showed haemorrhaging in the dermis (Figure 4), whereas MSC‐treated wounds had increased collagen synthesis in the dermis and full coverage with an epidermal layer with minimal infiltration of immune cells. The efficacy of MSC transplantation was reliably improved at day 7 by the presence of SP, with an intact epidermal/ dermis layer and mature collagen fibres (Figure 4A and B). The recovery of the injured site was described according to epithelial coverage and granulation tissue thickness (Table 1). Immunostaining for the F4/80 (+) inflammatory macrophages showed that MSC transplantation lessened F4/80 (+) macrophage infiltration into injured sites, which was further lessened by the addition of SP (Figure S6).

**Table 1:** SP‐enhanced wound healing by accelerating epithelial migration, wound coverage and promoting formation of granulation tissue

Day post‐transplantation	Epithelial migration (mm)		Wound coverage (mm^2^)		Granulation tissue thickness (m)	
	Day 2	Day 7	Day 2	Day 7	Day 2	Day 7
Vehicle	3.72 ± 0.82	4.8 ± 2.4	10.86 ± 0.5	18.08 ± 1.8	589.6 ± 83.0	896.0 ± 203.6
MSC	4.51 ± 1.16	7.2 ± 1.1	15.97 ± 0.8	40.69 ± 0.4	654.9 ± 91.8	911.8 ± 129.8
MSC + SP	4.89 ± 1.92	7.9 ± 0.2	18.09 ± 1.5	48.99 ± 0.04	861.9 ± 164	990.6 ± 171.4

Note

Wound area was analysed based on the histological analysis. Epithelial migration was measured from wound edge to the epithelium covered wound region. Wound coverage was expressed based on epithelial migration length and defect area. Granulation tissue formation was evaluated based on the invasion of endothelial cells, influx of fibroblasts, collagen deposition and accumulation of additional macrophages. The data are represented as mean ± SD of three independent experiments. N = 8 for each group.

This data suggests that the late MSC transplantation accelerated wound healing by stimulating epidermal migration and granulation tissue regeneration while suppressing inflammation. Importantly, all outcomes from MSC transplantation were enhanced by co‐treatment with SP.

### 3.3 SP accelerates angiogenesis by MSCS at the wound site

**Figure 5:**
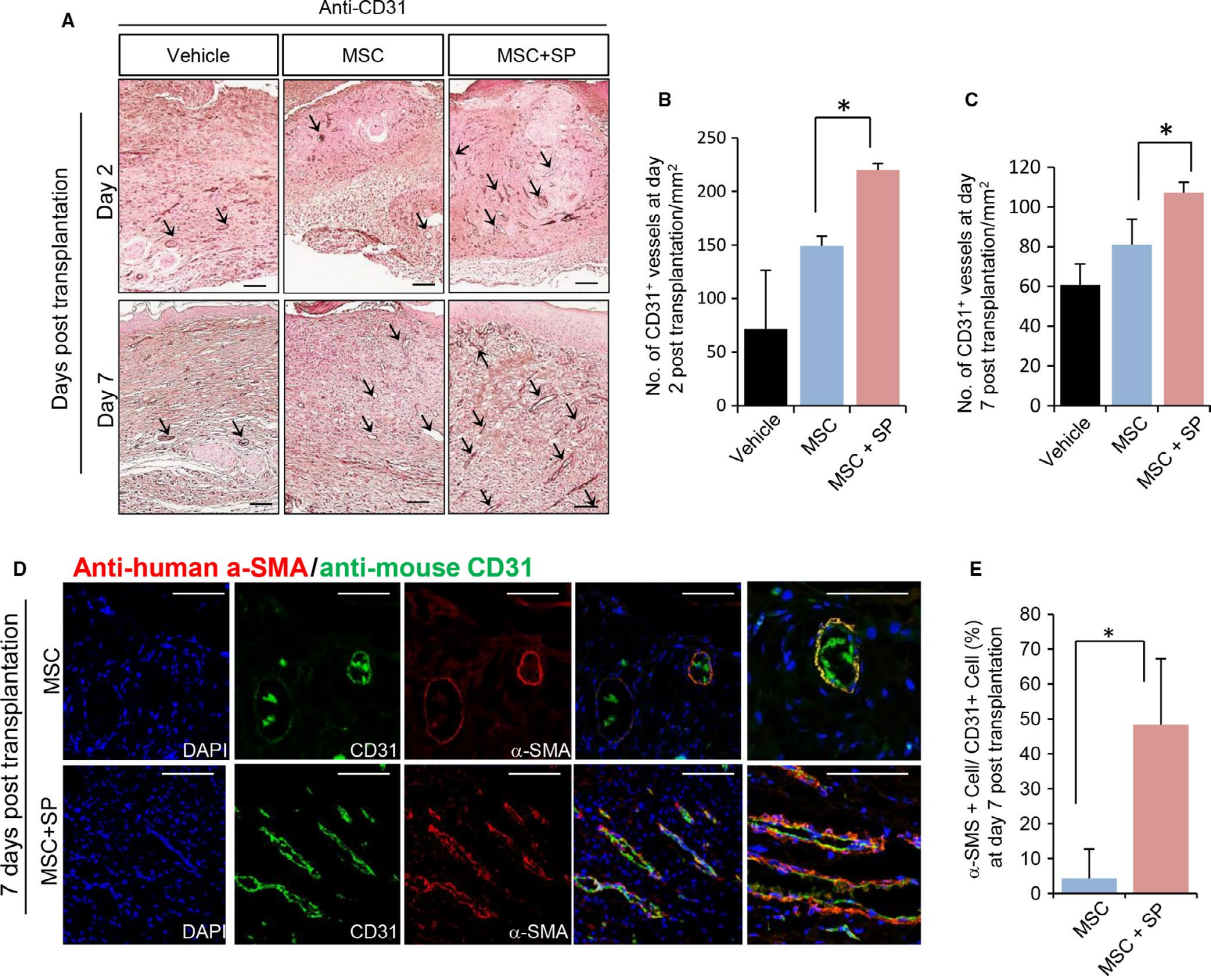
SP improves MSC‐induced angiogenesis at the wound site. Histological analysis of wounded tissue at day 7 post‐wound was performed. (A) Histological analysis of the vasculature was performed via staining for CD31 (B‐C) Quantification of CD31 (+) vasculature was performed at days 2 and 7 post‐transplantation. (D) Immunofluorescence staining of skin tissues with anti‐human α‐SMA and anti‐mouse CD31 to detect the localization of transplanted MSCs at the host vasculature. (E) Transplanted human α‐SMA (+) MSCs‐encircled vessels of total mouse CD31 (+) vessels were quantified. α‐SMA (+) human MSCs was shown in red and CD31 (+) mouse vascular endothelial cells are shown in green. Values of *P* < .05 were considered statistically significant (*P < .05). The data are represented as mean ± SD of three independent experiments. N = 8 for each group. Scale bar: 100 μm

Next, we explored whether MSC + SP‐induced fast wound healing was accompanied by neovascularization. Angiogenesis at the wound site was determined by immunohistochemical staining for CD31, specific for mouse vascular endothelial cells and vascular density was quantified by counting CD31 + vasculature per area (Figure 5A‐C). Compared to vehicle‐treated wounds, MSC‐transplanted wounds displayed a higher density of CD31 + vasculature, but the difference was not statistically significant. In contrast, MSC + SP‐transplantation promoted significantly more angiogenesis than MSC transplantation alone (CD31 + vessel/mm^2^, day 2 post‐transplantation, vehicle: 71.6 ± 54.9, MSC: 149.1.9 ± 9.2, MSC + SP: 220.3 ± 5.7, MSC vs. MSC + SP *P* < .05; day 7 post‐transplantation, vehicle: 60.69 ± 10.6, MSC: 81.1 ± 12.7, MSC + SP: 107.2 ± 5.3, MSC vs. MSC + SP, *P* < .05).

Among angiogenesis factors, SDF‐α plays a critical role in neovascularization. SP was observed to stimulate the production of SDF‐α from MSCs in vitro (Figure 1). As the transplantation of MSCs supplemented with SP augmented vascular density, expression of SDF‐1α within the wound site was likely to be elevated. As expected, SDF‐1α expression in the vehicle‐treated and MSC‐transplanted wounds was seldom detected, but the combination of MSCs and SP led to an SDF‐1α‐enriched wound environment (Figure S7).

These results suggest the possibility that SP remotely contributes to neovascularization by MSCs through the induction of angiogenic factors, such as SDF‐1α, within the wound site.

### 3.4 SP contributes to incorporation of transplanted MSCS into the host vasculature

Pericyte attachment and migration require PDGF‐BB and TGF‐β signalling. SP stimulates TGF‐β^23^, ^41^ and PDGF‐BB secretion (Figure 1C) in the MSCs. Accordingly, we anticipated that the combination of MSC and SP would result in the formation of pericyte‐encircled vasculature by transplanted MSCs. Mouse vasculature was identified through immunostaining with mouse CD31‐specific antibody (stained in green), and transplanted human MSCs were detected using anti‐human alpha‐smooth muscle actin antibody (α‐SMA, stained in red). The specificity of each antibody was determined by staining mouse skin with anti‐human α‐SMA antibody or human MSCs with anti‐mouse CD31 antibody (Figure S8). In the absence of SP, transplanted MSCs were seldom detected in wound sites (Figure 5D). Meanwhile, in the presence of SP, α‐SMA + human MSCs encircled CD31 + mouse vasculature as pericytes, and perceptible level of human MSCs were detected in the wound bed (Figure 5D). The proportion of α‐SMA + human MSCs that encircled CD31 + mouse vasculature was quantitatively analysed (Figure 5E). The MSC + SP‐transplanted wounds had approximately fourfold more α‐SMA + cell‐encircled vessels than the MSC‐transplanted wounds (MSC: 4.33 ± 8.4%, MSC + SP: 48.33 ± 18.9%, MSC vs. MSC + SP *P* < .05).

Collectively, these results imply that SP induces the formation of mature vessels by promoting the incorporation of transplanted MSCs into newly forming vessels as pericytes.

### 3.5 PDGF‐PDFGR signalling in human MSCS is closely associated with the engagement of transplanted cells during neovascularization

PDGF‐BB is known to be secreted from activated macrophages as well as endothelial cells and binds to the PDGF receptor (PDGFR) in pericytes to control migration and attachment of pericytes in the vasculature. In the presence of SP, transplanted MSCs were attached to the host vasculature as pericytes, implying the activation of PDGF‐BB/ PDGFR signalling in human MSCs.

**Figure 6:**
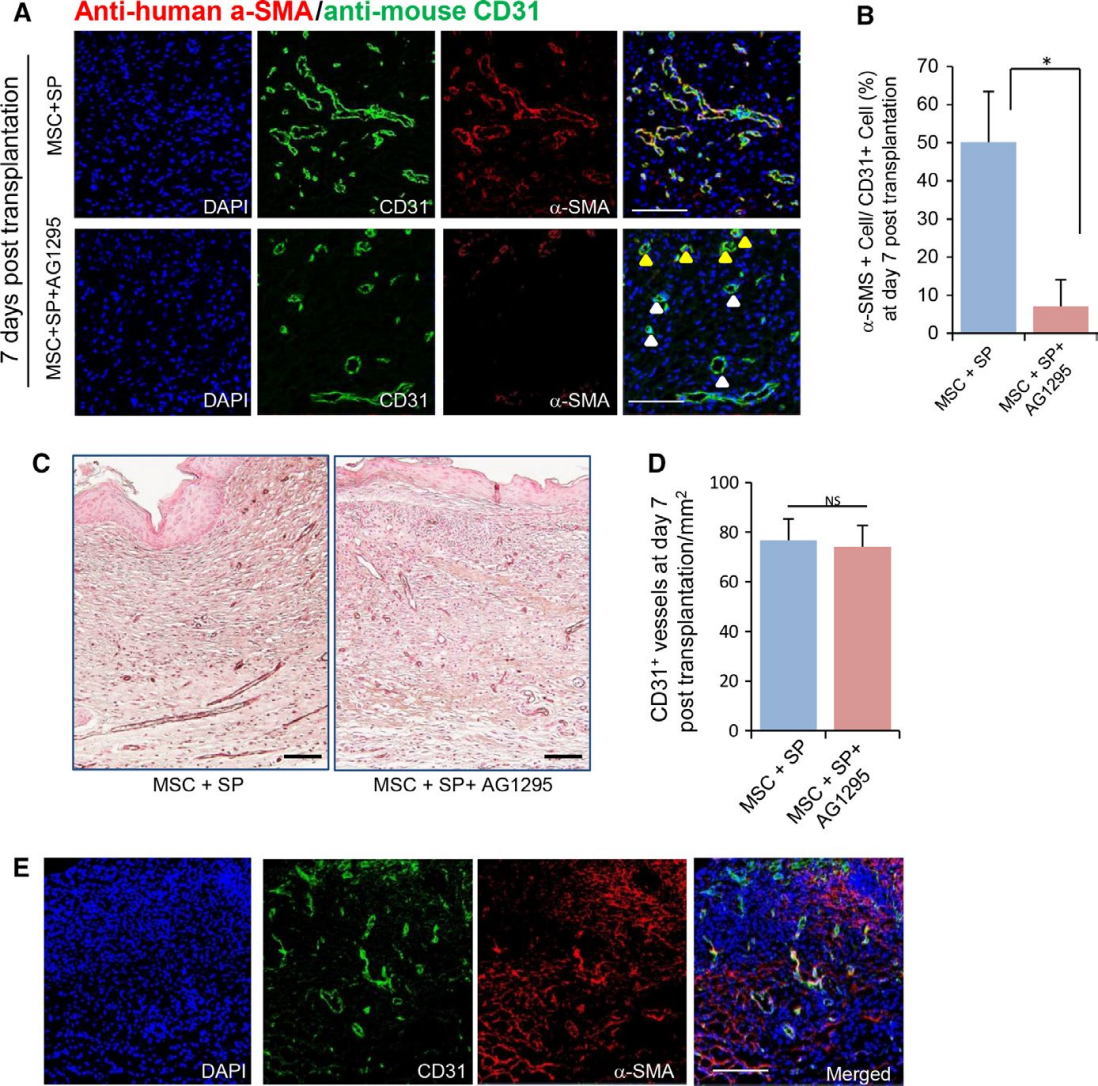
SP promotes the coverage of vasculature by transplanted MSCs via PDGF‐PDGFR signalling. (A) AG1295 was added to MSCs with SP to inhibit PDGF‐PDGFR signalling. Immunofluorescence staining of skin tissues with anti‐human α‐SMA and anti‐mouse CD31 to detect localization of transplanted MSCs in the host vasculature. (B) Transplanted human α‐SMA (+) MSCs encircling vessels of total mouse CD31 (+) vessels were quantified. (C) Histological analysis of the vasculature was performed via staining for CD31 (D) Quantification of CD31 (+) vasculature was performed at 7 post‐transplantation. (E) Immunofluorescence staining of skin tissues with anti‐human α‐SMA and anti‐mouse CD31 in AG1295‐treated mice. Values of *P* < .05 were considered statistically significant (*P < .05). The data are represented as mean ± SD of three independent experiments. N = 8 for each group. Scale bar100 μm. Yellow arrow head: Transplanted human α‐SMA (+) MSCs‐ encircled vessels. White arrow head: vessel without transplanted human α‐SMA (+) MSCs

To determine whether the attachment of transplanted MSCs to mouse vasculature was mediated by PDGF‐PDGFR signalling, MSCs supplemented with SP were treated with a PDGFR protein kinase inhibitor, AG1295, prior to transplantation. At day 7 post‐transplantation, vascular structure at the wound site was analysed by immunofluorescence staining. Treatment of AG1295 reduced the overall proportion of α‐SMA + human cell‐covered vasculature compared to the vascular structure in MSC + SP‐treated wounds (Figure 6A and B, MSC + SP: 50.12 ± 13.3%, MSC + SP+AG1295: 7.12 ± 7%, MSC vs. MSC + SP, *P* < .05). In addition, AG1295 treatment did not affect the total vessel density at all (Figure 6C and D), indicating that the blockage of PDGF‐PDGFR signalling did not influence quantity of vessel.

Consistent to previous reports, PDGF‐BB is capable of stimulating repopulation of MSC (Figure S9). Thus, the lack of human MSC‐covered vasculature in AG1295‐treated group is likely due to the reduced survival of transplanted MSCs. However, we found that the transplanted MSCs were scattered in the dermis instead of covering the host vasculature in AG1295‐treated mice (Figure 6E). Thus, the lowered density of human MSC‐covered vasculature in AG1296 treated mouse is not due to the decrease in survival of transplanted cells but the lack of attachment to the host vasculature.

This result suggests that the activation of PDGF/PDGFR signalling in transplanted human MSCs is essential for the attachment of MSCs in the vasculature.

### 3.6 Transplantation of MSCS with SP provokes suppression of systemic inflammation

**Figure 7:**
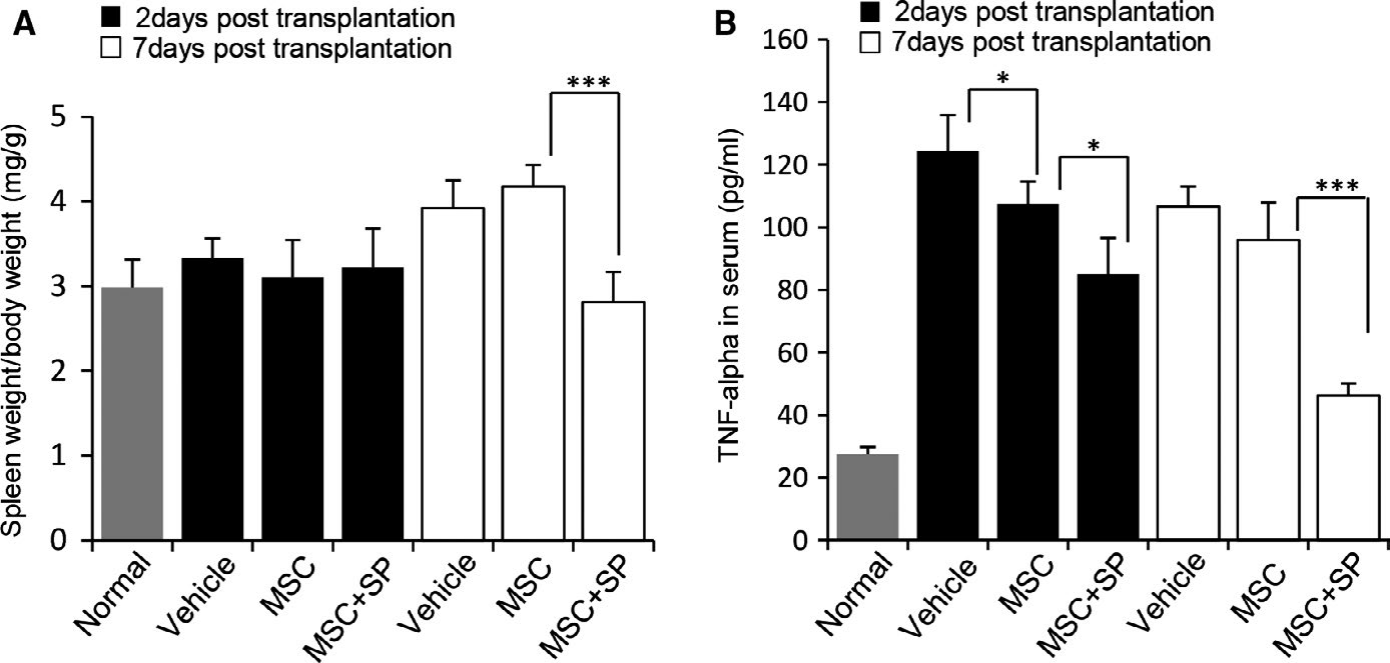
SP systematically improved MSC‐mediated immunosuppression. (A) As an indicator for systemic inflammation, spleen weight per body weight was assessed at days 2 and 7 post‐MSC transplantation. (B) Quantification of TNF‐α in serum was carried out by ELISA at days 2 and 7 post‐MSC transplantation. Values of *P* < .05 were considered statistically significant (*P < .05, **P < .01, ***P < .001). The data are represented as mean ± SD of three independent experiments. N = 8 for each group

Full‐thickness cutaneous wounds can trigger both systemic and local inflammation. To determine whether MSCs and SP affected systemic inflammation during wound healing, spleen weight and serum cytokines, indicators of the systemic inflammation, were analysed.^31^ Spleen size was not different by day 5 post‐wound (day 2 post‐transplantation), but enlargement of the spleen owing to the wound became evident at day 10 post‐wound (day 7 post‐transplantation). Contrary to our expectations, MSC transplantation did not block spleen enlargement, whereas the combination of MSC and SP markedly inhibited enlargement of the spleen, maintaining a size similar to that of the normal (Figure 7A). Analysis of the white pulp area in the spleen, which consists of germinal centres and peripheral T‐cell compartments, indicated that the MSC with SP interrupted the expansion of the white pulp area in response to the wound, whereas MSC transplantation alone was unable to do so (Figure S10). TNF‐α, a cytokine representative of inflammation, was also quantitatively assessed using ELISA. The level of TNF‐α in the serum was remarkably elevated because of the wound from day 2 post‐transplantation in vehicle‐treated mice. MSC transplantation somewhat reduced TNF‐α levels at day 2 post‐transplantation, but this effect was not maintained by day 7 post‐transplantation. In contrast, MSC with SP reduced TNF‐α levels from day 2 post‐transplantation, and the lower levels were sustained at day 7 post‐transplantation (Figure 7B, day 2 post‐transplantation, normal: 27.4 ± 2.4 pg/ml, vehicle: 124.3 ± 11.5 pg/ml, MSC: 107.5 ± 7.1 pg/ml, MSC + SP: 85.1 ± 11.4 pg/ml, MSC vs. MSC + SP *P* < .05; day 7 post‐transplantation, vehicle: 106.6 ± 6.4 pg/ml, MSC: 95.9 ± 12.1 pg/ml, MSC + SP: 46.3 ± 3.8 pg/ml, MSC vs. MSC + SP *P* < .001).

These data reveal that the addition of SP provokes the suppression of systemic inflammatory responses owing to the cutaneous wounds following transplantation of late passage MSCs, contributing to the faster repair.

## 4 DISCUSSION

Transplantation of ex vivo‐expanded MSCs improves tissue repair in vascular and inflammatory diseases, including infarcted hearts.^5^, ^6^, ^7^, ^8^, ^9^ However, ex vivo culture causes cellular senescence and reduces the release of paracrine factors in MSCs, damping the effect of MSC therapy. Moreover, as stem cells are transplanted into an inflammatory site and account for apoptotic sign as DAMP, it may be difficult for transplanted cells to survive for long periods. Accordingly, strategies are needed to enhance cellular survival and the activity of MSCs. As a way to improve the efficacy of MSC transplantation, we employed SP, a small endogenous peptide known to modulate vascular activity by affecting vasodilation and endothelial cell proliferation^42^ and provoke the secretion of angiogenic factors, including VEGF, in stem cells.^29^ In addition, SP was reported to restore the immunosuppressive function of late‐passage MSCs by elevating TGF‐β1 secretion.^23^ Based on these actions of SP, we hypothesized that the addition of SP can exert a supplemental effect on MSC therapy and facilitate MSC‐mediated healing.

We confirmed the deficiency of paracrine factor production in late‐passage MSCs and SP‐induced restoration of paracrine potential in senescent MSCs in vitro. To check the effect of SP on the efficacy of late‐passage MSCs in vivo, we transplanted late‐passage MSCs with insufficient paracrine/angiogenic potential, together with SP. Although transplantation of late‐passage MSCs yielded faster cutaneous wound healing than treatment with vehicle alone, the combination of late‐passage MSCs and SP resulted in further improvements in wound closure with well‐organized tissue regeneration, compared to MSCs alone.

Neovascularization is chiefly mediated by angiogenic factors, including SDF‐1α, PDGF‐BB and VEGF. The secretion of these factors in MSCs was elevated by SP treatment in vitro, suggesting that SP is indirectly involved in MSC‐mediated neovascularization in vivo. This study proved that SP could enhance MSC‐mediated neovascularization, possibly by providing an SDF‐1α‐enriched wound bed. Interestingly, transplanted MSCs encircled the host vasculature as pericytes. This finding demonstrates the direct engagement of transplanted MSCs in vascular formations, which might be mediated by SP.

The coverage of vasculature by pericytes is modulated by PDGF‐PDGFR signalling. The blockage of PDGF‐PDGFR signalling during the transplantation of MSCs with SP decreased the amount of vessels covered with transplanted MSCs in vivo, even in the presence of SP. In this case, transplanted cells were observed at the dermis, not attaching to host vessels. This means that PDGF‐PDGFR signalling is involved in the migration and attachment of MSCs to the vasculature as pericytes.

Because the PDGF‐BB amino acid sequences of human and mouse have high homology, the cellular source of PBGF‐BB may be unclear. Considering that the source of PDGF‐BB is chiefly macrophages and endothelial cells and we transplanted MSCs and SP in the inflammatory phase which lacks endogenous vessels, activated macrophages that contacted with SP were inferred to be the one of sources of PDGF‐BB for MSCs, except for transplanted human MSCs. However, SP could not promote the secretion of PDGF‐BB from activated mouse macrophages in vitro (Figure S11). Thus, it could be surmised that the PDGF‐BB that binds to the PDGFR of human MSCs is obtained from transplanted human MSCs affected by SP and that human PDGF‐BB functions via autocrine activation to promote the attachment of transplanted MSCs to the vascular surface.

Cutaneous wounds were accompanied by systemic inflammation. These inflammatory responses were barely suppressed by late‐passage MSCs, likely due to the weakened activity of senescent MSCs. However, the combination of MSCs and SP alleviated systemic inflammation by reducing spleen size and the serum level of TNF‐α. This phenomenon led to improved wound healing.

Although topical treatment with SP has been shown to stimulate skin wound healing,^30^ we did not observe the same effect in our study, possibly because we only administered SP once (Figure S2) and SP is degraded rapidly at wound site. The previous study involving the local treatment with SP for cutaneous wound healing emphasized the need for repeated daily treatment with SP for complete healing. Thus, we attribute the MSCs + SP‐induced tissue repair seen in this study to the SP‐enhanced therapeutic potential of MSCs rather than the direct effect of SP.

SP‐enhanced therapeutic potential of MSCs was occurred by restoring paracrine action of BM MSC in this study. The exact mechanism of SP for blockage of cellular senescence is unclear. However, senescence of MSC was explored broadly. Notably, RAP1/NFkb signalling pathway was proved to be chiefly involved in immune modulatory function of MSC.^43^, ^44^ ERBB4 can rejuvenate aged MSC to promote for tissue repair through PI3K/ Akt and MAPK/Erk pathways.^45^, ^46^ SP could activate NFkb signalling and also active Akt/ Erk signalling to suppress cellular apoptosis.^29^, ^47^, ^48^, ^49^ Considering previous reports, the effect of SP on inhibition of BM MSC senescence may be ratiocinated and the related mechanism may be uncovered. This should be explored for further study.

The therapeutic effects of systemically administered SP have been reported in diverse diseases. However, to the best of our knowledge, this is the first study to establish the supplemental effects of SP on stem cell therapy in vivo. The combination of SP and MSCs will solve some of the challenges underlying MSC therapeutics. The safety of SP as a drug was recently documented^50^ and, so, administration of SP with MSCs is not expected to cause toxicity.

Collectively, our findings support the development of SP as a supplemental agent for MSC therapy in inflammatory or ischaemic diseases. Further detailed study of the effects of SP supplementation on other stem cell subsets in a variety of diseases is planned.

## 5 CONCLUSIONS

Our findings demonstrated that the supplementation of SP improved the efficacy of MSCs.

## CONFLICT OF INTEREST

The authors have no conflict of interest to declare.

## AUTHOR CONTRIBUTIONS

**Hyun Sook Hong:** Conceptualization (lead); Data curation (lead); Funding acquisition (lead); Project administration (lead); Supervision (lead); Writing‐original draft (lead); Writing‐review & editing (lead). **Suna Kim:** Conceptualization (supporting); Data curation (supporting); Formal analysis (supporting); Methodology (equal). **Yinji Jin:** Data curation (supporting); Investigation (supporting). **Youngsook Son:** Conceptualization (equal); Supervision (equal); Writing‐original draft (equal).

## Supporting information

Supplementary Material

## Data Availability

The dataset used in this study is available from the corresponding author on reasonable request.

## References

[1] Chen L , Tredget EE , Wu PYG , et al. Paracrine factors of mesenchymal stem cells recruit macrophages and endothelial lineage cells and enhance wound healing. PLoS One. 2008;3:e1886. 18382669 10.1371/journal.pone.0001886 PMC2270908

[2] Satoh H , Kishi K , Tanaka T , et al. Transplanted mesenchymal stem cells are effective for skin regeneration in acute cutaneous wounds. Cell Transplant. 2004;13:405‐412. 15468682 10.3727/000000004783983765

[3] Pittenger MF , Mackay AM , Beck SC , et al. Multilineage potential of adult human mesenchymal stem cells. Science. 1999;284:143‐147. 10102814 10.1126/science.284.5411.143

[4] Mackenzie TC , Flake AW . Human mesenchymal stem cells persist, demonstrate site‐specific multipotential differentiation, and are present in sites of wound healing and tissue regeneration after transplantation into fetal sheep. Blood Cells Mol Dis. 2001;27:601‐604. 11482873 10.1006/bcmd.2001.0424

[5] Xue L , Xu YB , Xie JL . Effects of human bone marrow mesenchymal stem cells on burn injury healing in a mouse model. Int J Clin Exp Pathol. 2013;6:1327‐1336. 23826413 PMC3693197

[6] Lan Y , Kodati S , Lee HS , et al. Kinetics and function of mesenchymal stem cells in corneal injury. Invest Ophthalmol Vis Sci. 2012;53:3638‐3644. 22562508 10.1167/iovs.11-9311

[7] Liu L , Yu Y , Hou Y , et al. Human umbilical cord mesenchymal stem cells transplantation promotes cutaneous wound healing of severe burned rates. PLoS One. 2014;9:e88348. 24586314 10.1371/journal.pone.0088348 PMC3930522

[8] Semedo P , Correa‐Costa M , Antonio Cenedeze M , et al. Mesenchymal stem cells attenuate renal fibrosis through immune modulation and remodeling properties in a rat remnant kidney model. Stem Cell. 2009;27:3063‐3073. 10.1002/stem.214 19750536

[9] Bian S , Zhang L , Duan L , et al. Extracellular vesicles derived from human bone marrow mesenchymal stem cells promote angiogenesis in a rat myocardial infarction model. J Mol Med. 2014;92:387‐397. 24337504 10.1007/s00109-013-1110-5

[10] El‐denshary ESM , Rashed LA , Elhussiny M . Immunosuppressive effects of mesenchymal stem cells versus corticosteroid in experimental model of arthritis. Clin Exp Pharmacol. 2015;S5:3. 10.1007/s10238-013-0248-3 23990050

[11] Meisel R , Zibert A , Laryea M . Human bone marrow stromal cells inhibit allogeneic T‐cell responses by indoleamine 2,3‐dioxygenase‐mediated tryptophan degradation. Blood. 2004;103:4619‐4621. 15001472 10.1182/blood-2003-11-3909

[12] Németh K , Leelahavanichkul A , Yuen PS . Bone marrow stromal cells attenuate sepsis via prostaglandin E (2)‐dependent reprogramming of host macrophages to increase their interleukin‐10 production. Nat Med. 2009;15:42‐49. 19098906 10.1038/nm.1905 PMC2706487

[13] Ghannam S , Bouffi C , Djouad F . Immunosuppression by mesenchymal stem cells: mechanisms and clinical applications. Stem Cell Res Ther. 2010;1:2‐8. 20504283 10.1186/scrt2 PMC2873698

[14] Liu H , Lu K , MacAry PA . Soluble molecules are key in maintaining the immnunomodulatory activity of murine mesenchymal stromal cells. J Cell Sci. 2012;125:200‐208. 22250196 10.1242/jcs.093070

[15] Newman RE , Yoo D , LeRoux MA . Treatment of inflammatory diseases with mesenchymal stem cells. Inflamm Allergy Drug Targets. 2009;8:110‐123. 19530993 10.2174/187152809788462635

[16] Wu Y , Chen L , Scott PG . Mesenchymal stem cells enhance wound healing through differentiation and angiogenesis. Stem Cells. 2007;25:2648‐2659. 17615264 10.1634/stemcells.2007-0226

[17] Wu Y , Chen L , Scott PG . Mesenchymal stem cells are recruited into wounded skin and contribute to wound repair by transdifferentiation into multiple skin cell type. J Immunol. 2008;180:2581‐2587. 18250469 10.4049/jimmunol.180.4.2581

[18] Crisan M , Yap S , Casteilla L , et al. A perivascular origin for mesenchymal stem cells in multiple human organs. Cell Stem Cell. 2008;3:301‐313. 18786417 10.1016/j.stem.2008.07.003

[19] Schellenberg A , Lin Q , Schüler H . Replicative senescence of mesenchymal stem cells causes DNA‐methylation changes which correlate with repressive histone marks. Aging (Albany NY). 2011;3:873‐888. 22025769 10.18632/aging.100391 PMC3227452

[20] Wagner W , Horn P , Castoldi M , et al. Replicative senescence of mesenchymal stem cells: a continuous and organized process. PLoS One. 2008;3:e2213. 18493317 10.1371/journal.pone.0002213 PMC2374903

[21] Samsonraj RM , Raghunath M , Hui JH . Telomere length analysis of human mesenchymal stem cells by quantitative PCR. Gene. 2013;519:348‐355. 23380569 10.1016/j.gene.2013.01.039

[22] Yu KR , Lee JY , Kim HS , et al. p38 MAPK‐Mediated Alteration of COX‐2/PGE2 regulates Immunomodulatory Properties in Human Mesenchymal Stem Cell Aging. PLoS One. 2014;9:e102426. 25090227 10.1371/journal.pone.0102426 PMC4121064

[23] Jin Y , Hong HS , Son Y . Substance p enhances mesenchymal stem cell mediated immune modulation. Cytokine. 2015;71:145‐153. 25461392 10.1016/j.cyto.2014.10.003

[24] Saini U , Gumina RJ , Wolfe B , et al. Preconditioning mesenchymal stem cells with caspase inhibition and hyperoxia prior to hypoxia exposure increases cell proliferation. J Cell Biochem. 2013;114:2612‐2623. 23794477 10.1002/jcb.24609 PMC4017598

[25] Wang L , Pasha Z , Wang S , et al. Protein kinase G1 α overexpression increases stem cell survival and cardiac function after myocardial infarction. PLoS One. 2013;8:e60087. 23536905 10.1371/journal.pone.0060087 PMC3607603

[26] Rameshwar P , Gascon P . Substance P (SP) mediated production of stem cell factor and interleukin‐1 in bone marrow: potential autoregulatory role for these cytokines in SP receptor expression and induction. Blood. 1995;85:482‐490. 7541664

[27] Rameshwar P , Joshi DD , Yadav P , et al. Mimicry between neurokinin‐1 and fibronectin may explain the transport and stability of increased substance P immunoreactivity in patients with bone marrow fibrosis. Blood. 2001;97:3025‐3031. 11342427 10.1182/blood.v97.10.3025

[28] Yang CM , Hsiao LD , Chien CS , et al. Substance P‐induced activation of p42/44 mitogen‐activated protein kinase associated with cell proliferation in human tracheal smooth muscle cells. Cell Signal. 2002;14:913‐923. 12220617

[29] Hong HS , Lee J , Lee E , et al. A new role of substance P as an injury‐inducible messenger for mobilization of CD29+ stromal‐like cells. Nat Med. 2009;15:425‐435. 19270709 10.1038/nm.1909

[30] Kant V , Gopal A , Kumar D , et al. Topically applied substance P enhanced healing of open excision wound in rats. Eur J Pharmacol. 2013;715:345‐353. 23684543 10.1016/j.ejphar.2013.04.042

[31] Hong HS , Son Y . Substance P ameliorates collagen II‐induced arthritis in mice via suppression of the inflammatory response. Biochem Biophys Res Commun. 2014;453:179‐184. 25264193 10.1016/j.bbrc.2014.09.090

[32] Jiang MH , Lim JE , Chi GF , et al. Substance P reduces apoptotic cell death possibly by modulating the immune response at the early stage after spinal cord injury. NeuroReport. 2013;24:846‐851. 23995292 10.1097/WNR.0b013e3283650e3d

[33] Kang MH , Kim DY , Yi JY , et al. Substance‐P accelerates intestinal tissue regeneration after gamma irradiation‐induced damage. Wound Repair Regen. 2009;17:216‐223. 19320890 10.1111/j.1524-475X.2009.00456.x

[34] Salvucci O , Yao L , Villalba S , et al. Regulation of endothelial cell branching morphogenesis by endogenous chemokine stromal‐derived factor‐1. Blood. 2002;99:2703‐2711. 11929756 10.1182/blood.v99.8.2703

[35] Hammes HP , Lin J , Renner O , et al. Pericytes and the pathogenesis of diabetic retinopathy. Diabetes. 2002;51:3107‐3112. 12351455 10.2337/diabetes.51.10.3107

[36] Rucker HK , Wynder HJ , Thomas WE . Cellular mechanisms of CNS pericytes. Brain Res. Bull. 2000;51:363‐369. 10715555 10.1016/s0361-9230(99)00260-9

[37] Lindahl P , Johansson BR , Levéen P , et al. Pericyte loss and microaneurysm formation in PDGF‐B‐deficient mice. Science. 1997;277:242‐245. 9211853 10.1126/science.277.5323.242

[38] Soriano P . Abnormal kidney development and hematological disorders in PDGF beta‐receptor mutant mice. Genes Dev. 1994;8:1888‐1896. 7958864 10.1101/gad.8.16.1888

[39] Zhang JM , Feng FE , Wang QM , et al. Platelet‐Derived Growth Factor‐BB protects mesenchymal stem cells (MSCs) derived from immune thrombocytopenia patients against apoptosis and senescence and maintains MSC‐mediated immunosuppression. Stem Cell Transl Med. 2016;5:1631‐1643. 10.5966/sctm.2015-0360 PMC5189646 27471307

[40] Fierro F , Illmer T , Jing D , et al. Inhibition of platelet‐derived growth factor receptorβ by imatinib mesylate suppresses proliferation and alters differentiation of human mesenchymal stem cells in vitro. Cell Prolif. 2007;40:355‐366. 17531080 10.1111/j.1365-2184.2007.00438.x PMC6496321

[41] Hu D , Chen B , Zhu X , et al. Substance P up‐regulates the TGF‐beta 1 mRNA expression of human dermal fibroblasts in vitro. Zhonghua Zheng Xing Wai Ke Za Zhi. 2002;18:234‐236. 12382579

[42] Harrison S , Geppetti P . Substance p. Int. J. Biochem. Cell. Biol. 2001;33:555‐576. 11378438 10.1016/s1357-2725(01)00031-0

[43] Ding Y , Liang X , Zhang Y , et al. Rap1 deficiency‐provoked paracrine dysfunction impairs immunosuppressive potency of mesenchymal stem cells in allograft rejection of heart transplantation. Cell death dis. 2018;9:386. 29515165 10.1038/s41419-018-0414-3 PMC5842217

[44] Zhang Y , Chiu S , Liang X , et al. Rap1‐mediated nuclear factor‐kappaB (NF‐κB) activity regulates the paracrine capacity of mesenchymal stem cells in heart repair following infarction. Cell Death Discovery. 2015;1:15007. 27551443 10.1038/cddiscovery.2015.7 PMC4981000

[45] Liang X , Ding Y , Lin F , et al. Overexpression of ERBB4 rejuvenates aged mesenchymal stem cells and enhances angiogenesis via PI3K/AKT and MAPK/ERK pathways. FASEB J. 2019;33:4559‐4570. 30566395 10.1096/fj.201801690R

[46] Liang X , Ding Y , Zhang Y , et al. Activation of NRG1‐ERBB4 signaling potentiates mesenchymal stem cell‐mediated myocardial repairs following myocardial infarction. Cell Death Dis. 2015;6:e1765. 25996292 10.1038/cddis.2015.91 PMC4669719

[47] Lieb K , Fiebich BL , Berger M , et al. The neuropeptide substance P activates transcription factor NF‐kappa B and Kappa B‐dependent gene expression in human astrocytoma cells. J Immunol. 1997;159:4952‐4958. 9366421

[48] Seo EJ , Kim S , Hong HS . Substance‐p blocks ethanol‐induced hepatotoxicity. Life Sci. 2018;203:268‐275. 29730167 10.1016/j.lfs.2018.05.004

[49] DeFea KA , Vaughn ZD , O'Bryan EM , et al. The proliferative and antiapoptotic effects of substance P are facilitated by formation of a β‐arrestin‐dependent scaffolding complex. PNAS. 2000;97:11086‐11091. 10995467 10.1073/pnas.190276697 PMC27152

[50] Hong HS , Um JH , Son YS . Long‐term comparative study of Substance‐P with methylprednisolone on the development of osteoporosis. J Toxicol. Sci. 2014;39:391‐399. 24849674 10.2131/jts.39.391

