# Supplementary material for: Substance P enhances the therapeutic effect of MSCs by modulating their angiogenic potential
Source: J Cell Mol Med. 2020 Sep 28;24(21):12560–71. doi: 10.1111/jcmm.15804 (PMC7687016; doi:10.1111/jcmm.15804)
Supplement: Supplementary file 1 — Supplementary Material [file JCMM-24-12560-s001.pdf]

# Supplementary materials

**12 Figure, 1 table**

## Substance P enhances the therapeutic effect of MSCs by modulating their angiogenic potential

Hyun Sook Hong, Suna Kim, Yinji Jin, Yongsook Son

# Supplementary Figure 1

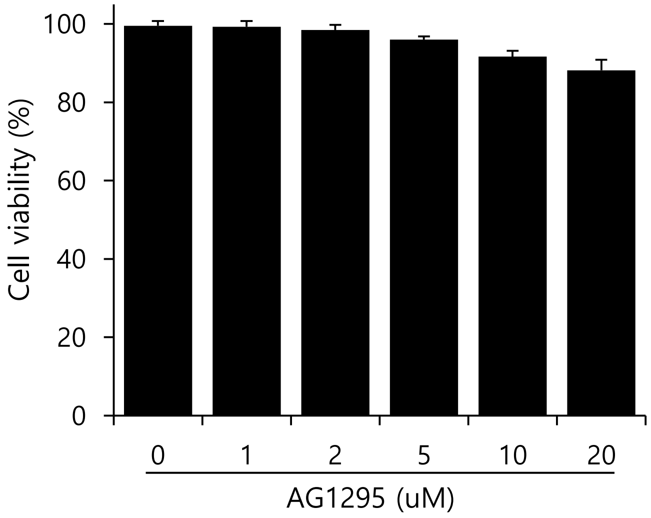


**Supplementary Figure 1. The evaluation of safety of AG1295 on MSCs**

AG1295 was treated to MSC in several doses to check the effect of AG1295 on cell viability, The data are expressed as the mean ± S.D.

# Supplementary Figure 2

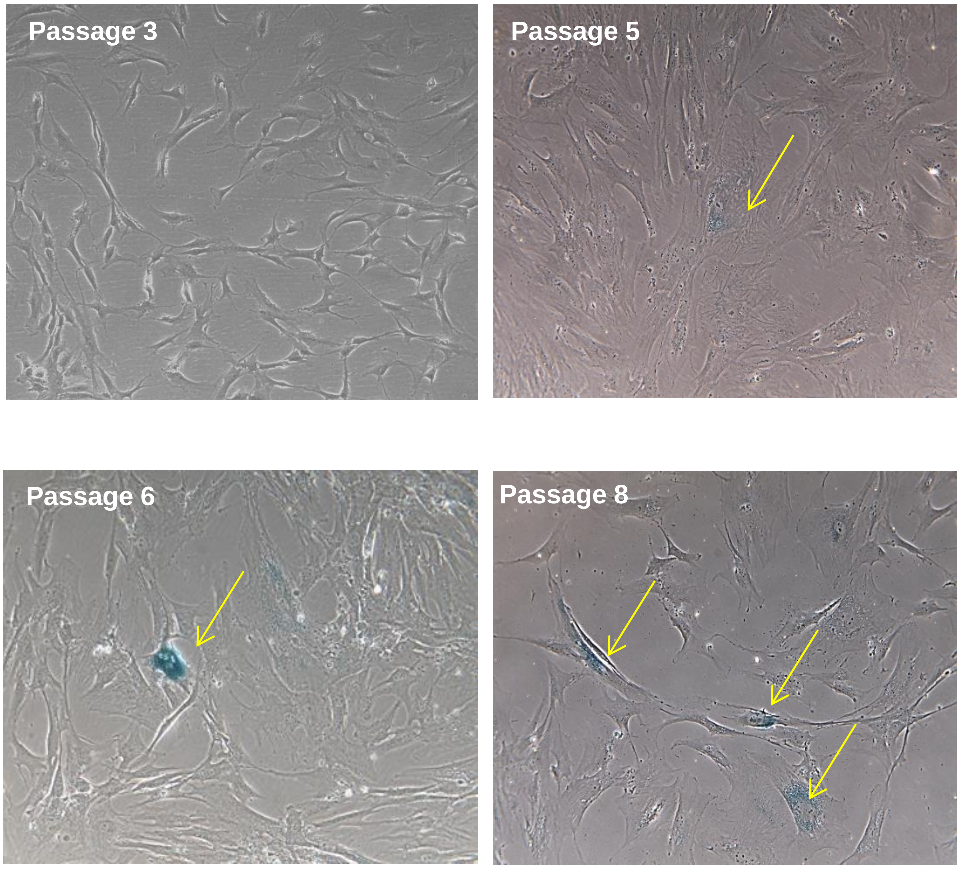


**Supplementary Figure 2. β-galactosidase staining of BM MSC in vitro**

MSCs were cultured in vitro and passage 3, 5, 6 and 8 of MSC were stained with beta-galactosidase to assess cellular senescence. Yellow arrow: Beta galactosidase+ cells

# Supplementary Figure 3

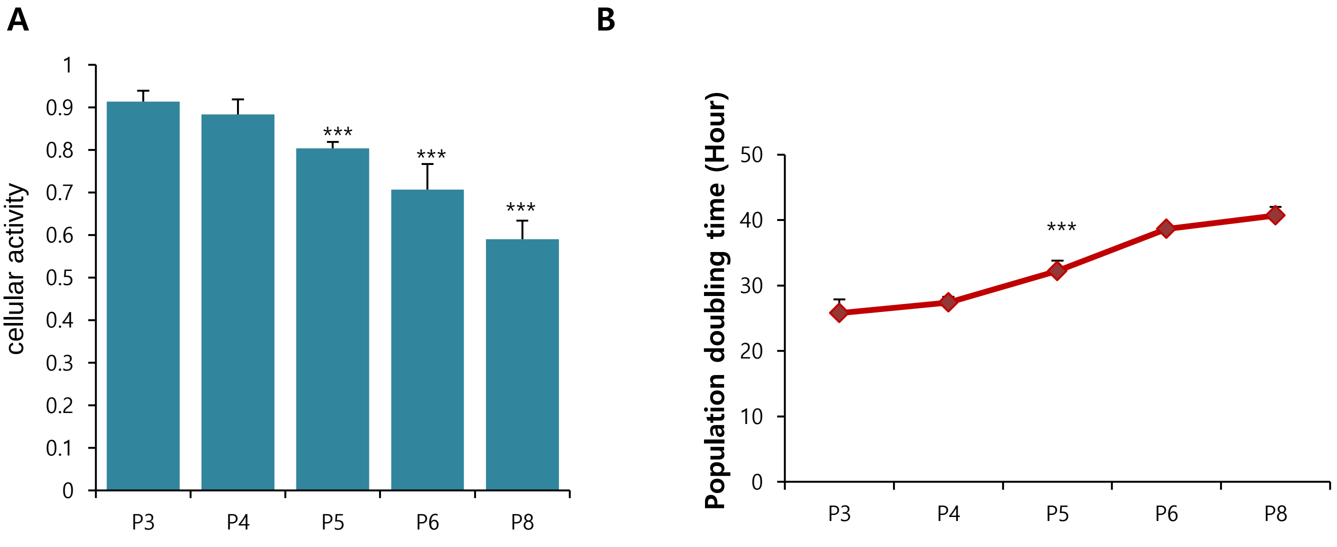


**Supplementary Figure 3. Population doubling time of BM MSC**

MSCs were cultured in vitro and analyzed for cellular activity and population doubling time. (A) WST-1 assay was performed. (B) population doubling time was analyzed from p3 to p8 BM MSC. Values of $p < 0.05$ were interpreted as statistically significant (***$p < 0.001$, vs P3 ). The data are expressed as the mean ± S.D of triplicate experiments.

# Supplementary Figure 4

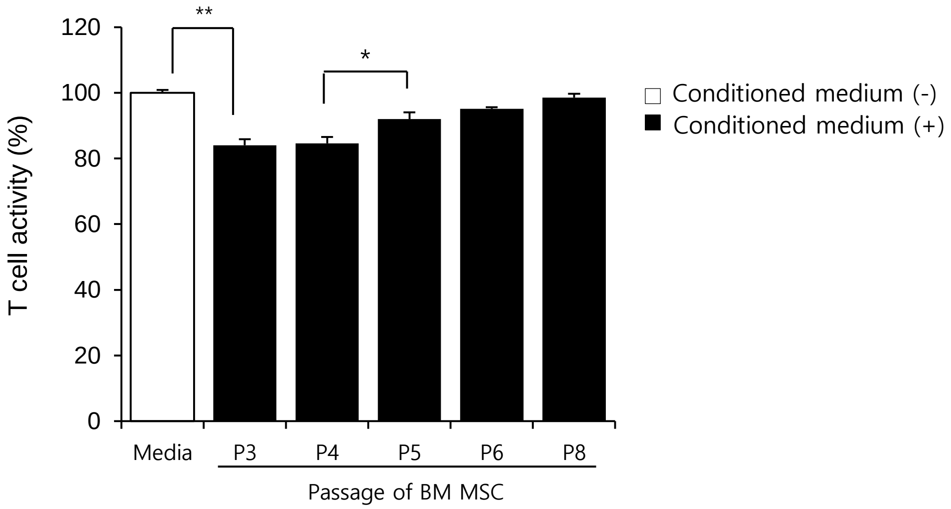


**Supplementary Figure 4. The immune suppressive effect of MSC on T cells**

MSCs were cultured in vitro and conditioned medium of MSC (p3-8) was added to Jurkat T cells. 24h later, WST-1 assay was carried out. T cell activity was represented as a percentage relative to activity of T cells with cell-free media. Values of $p < 0.05$ were interpreted as statistically significant (*$p < 0.05$, **$p < 0.01$). The data are expressed as the mean ± S.D of triplicate experiments

# Supplementary Figure 5

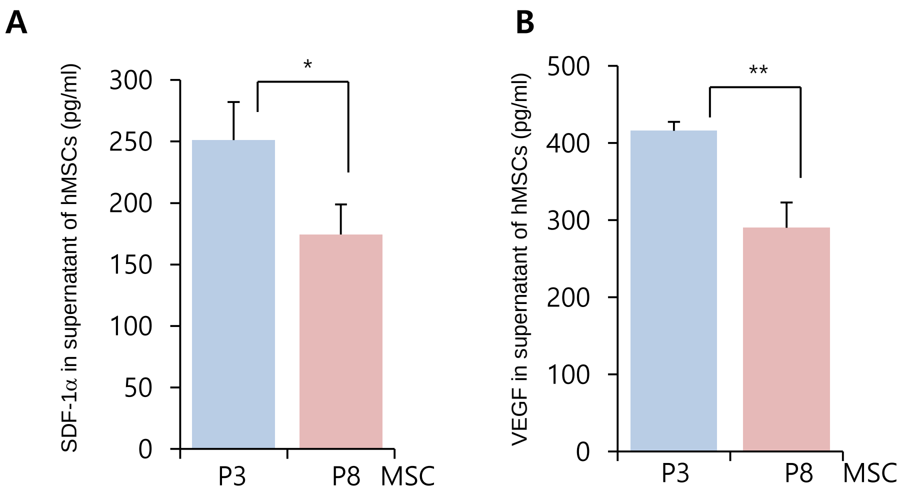


**Supplementary Figure 5. Ex vivo culture affects production of paracrine factor in MSCs**

MSCs were cultured in vitro and passage 3 and 8 of MSC were evaluated for secretion of SDF-α (A) and VEGF (B). Values of $p < 0.05$ were interpreted as statistically significant (*$p < 0.05$, **$p < 0.01$, ***$p < 0.001$). The data are expressed as the mean ± S.D of triplicate experiments

# Supplementary Figure 6

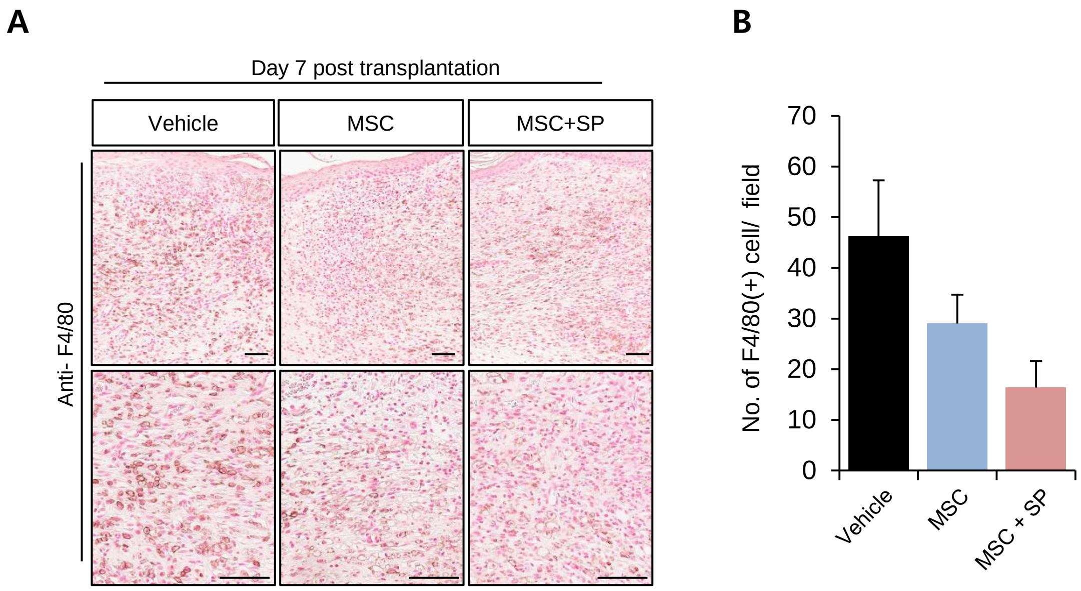


**Supplementary Figure 6. SP improves MSC-induced suppression of inflammation**
Histological analysis of wounded tissue at day 7 post-wound was performed. (A) Samples of 4-μm thickness were stained for F4/80 (+) macrophages at the wound bed. (B) F4/80 + cells were counted.

# Supplementary Figure 7

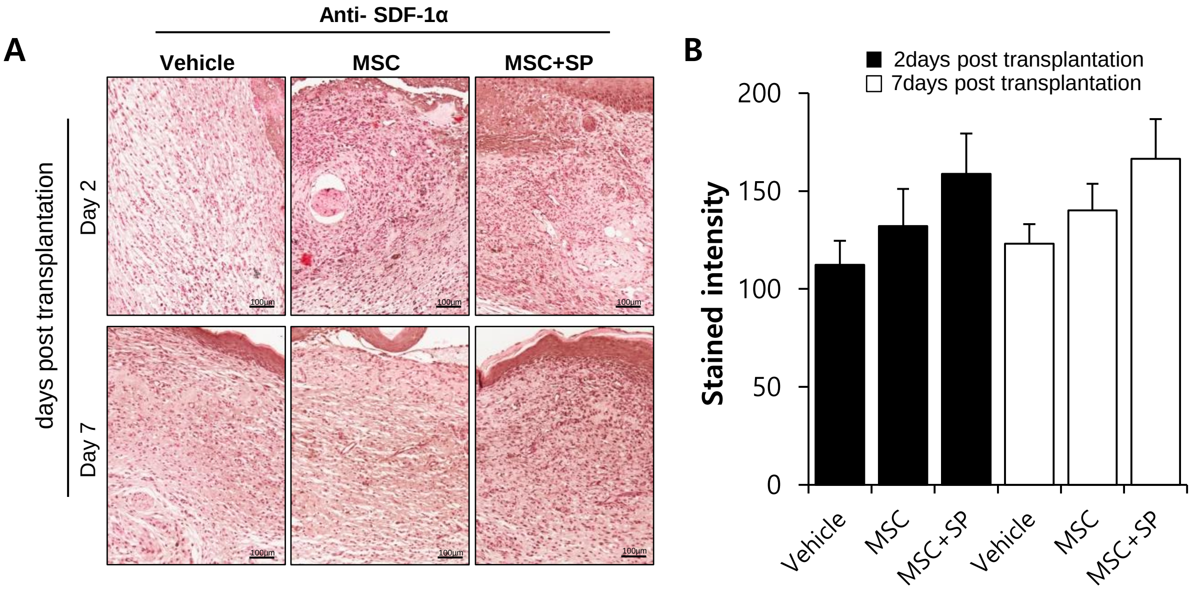


**Supplementary Figure 7. Immunohistochemical staining for SDF-1α at wound site.**

Histological analysis for wounded tissue was performed. The secretion of SDF-1 was detected at wound site at day 2 and 7 post MSC transplantation. n=6 for each group. Scale bar: 100 μm

# Supplementary Figure 8

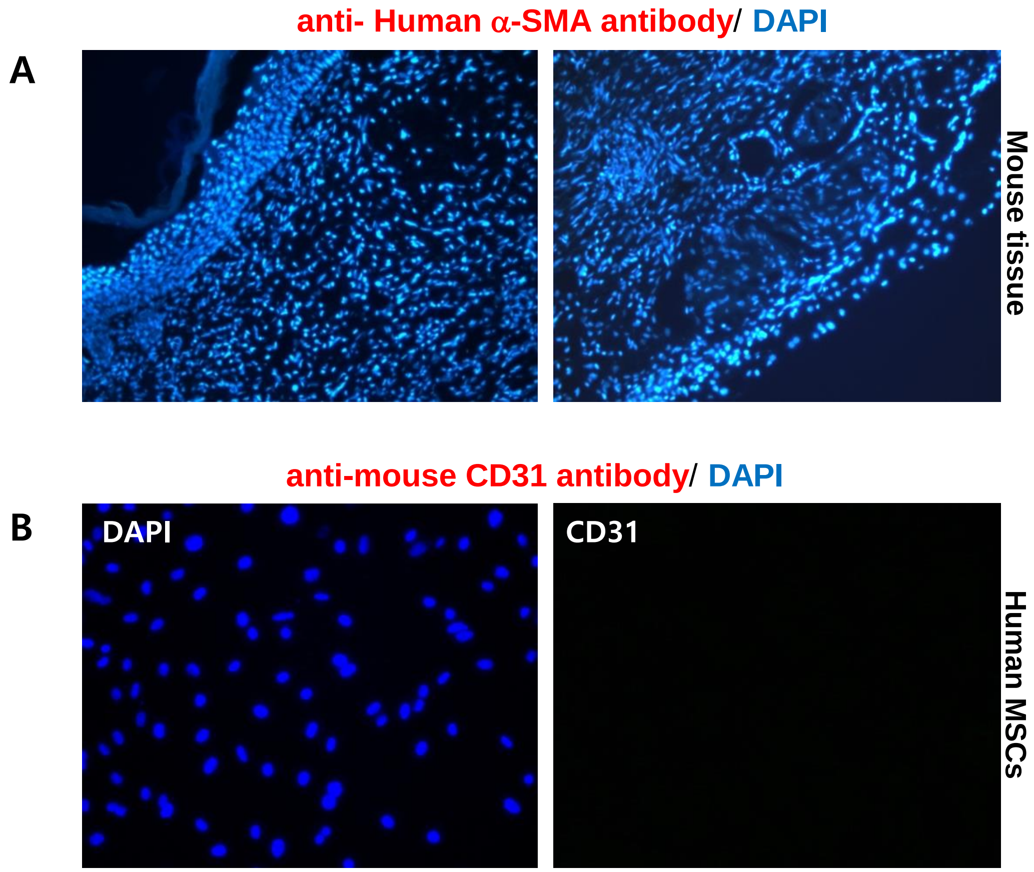


**Supplementary Figure 8. Examination for the reactivity of species-specific antibody.**

(A) The reactivity of human α-SMA antibody on mouse tissue was examined. No activity of α-SMA antibody was detected. (B) Human MSCs was stained with anti-mouse CD31 antibody.

# Supplementary Figure 9

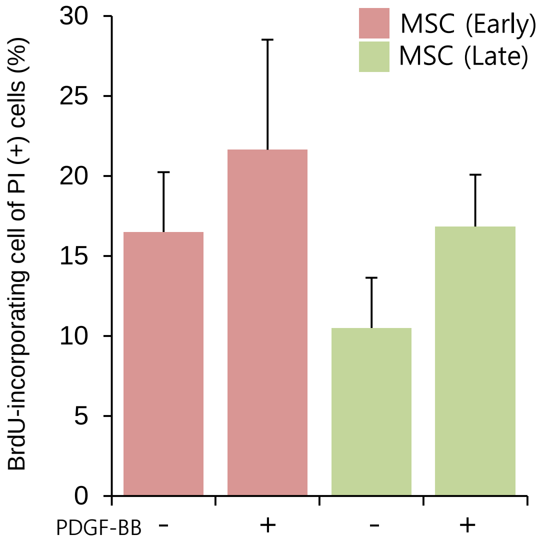


**Supplementary Figure 9. The effect of PDGF-BB on MSC proliferation in vitro.**

PDGF-BB (20ng/ml) was added to MSC at early and late passage and then 24h later, BrdU incorporation assay was performed to determine the proliferating cell pool. BrdU was treated for 3 h before cell fixation.
BrdU (+) cells was quantified by counting BrdU (+) cell of total PI (+) cells and expressing the percentage.

# Supplementary Figure 10

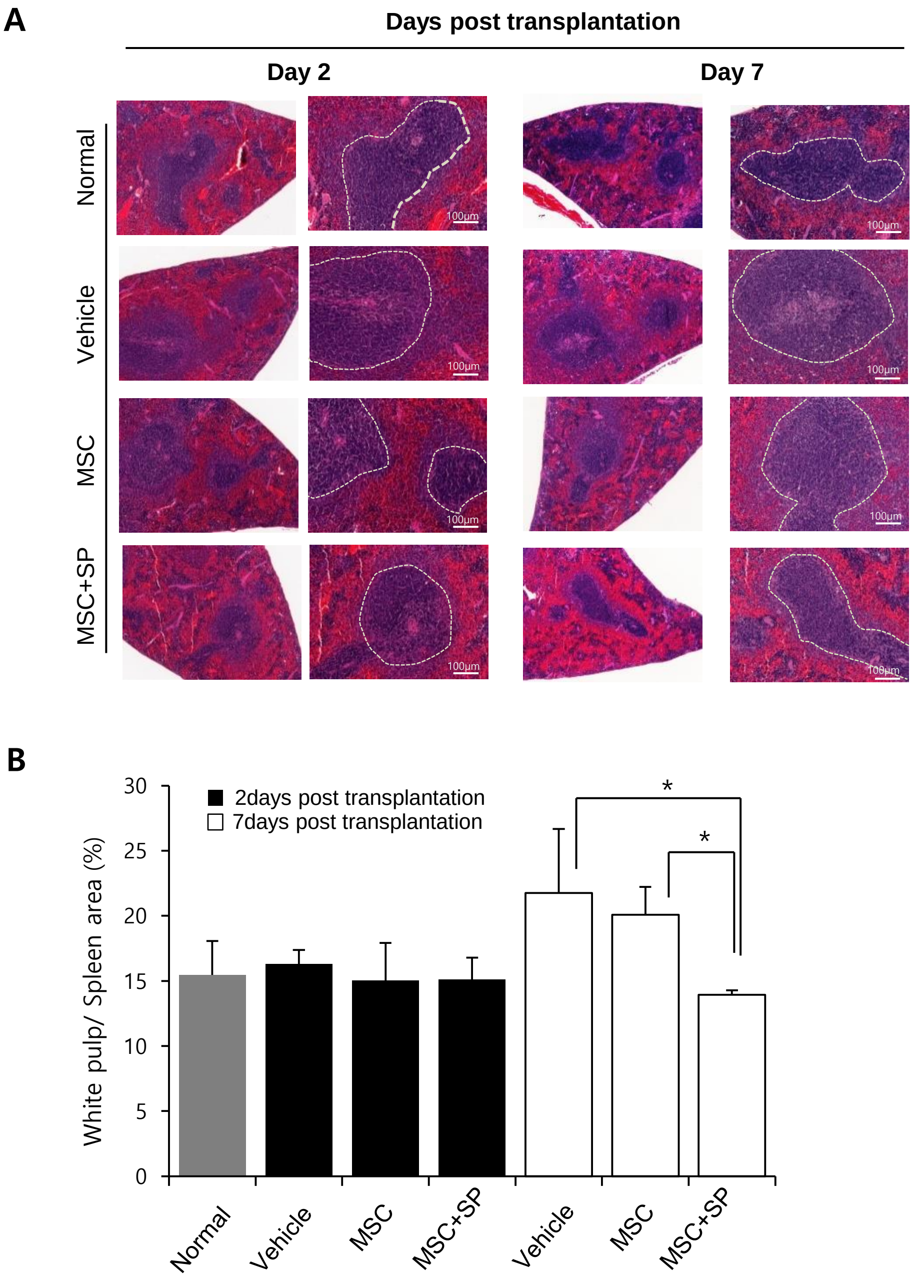


**Supplementary Figure 10. The histological analysis for spleen**

Histological analysis for spleen was performed. (A) Hematoxylin and eosin staining was carried out at wound site at day 2 and 7 post MSC transplantation. White dotted line: white pulp area. Scale bar: 100 μm (B) Quantitative analysis for white pulp of total spleen area. Values of $p < 0.05$ were interpreted as statistically significant (*$p < 0.05$, **$p < 0.01$, ***$p < 0.001$). The data are expressed as the mean ± S.D.

# Supplementary Figure 11

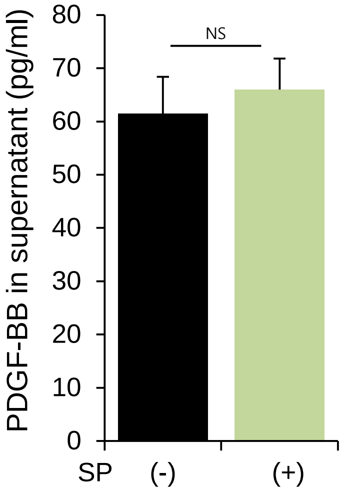


**Supplementary Figure 11. The effect of SP on PDGF-BB secretion in mouse macrophage *in vitro***

Raw 264.7, monocyte macrophage cell line, was incubated in 24 well with LPS and then, SP was added in concentration of final 100nM. 24h later, supernatant was collected and PDGF-BB was quantified by ELISA.

NS: Non significant

# Supplementary Figure 12

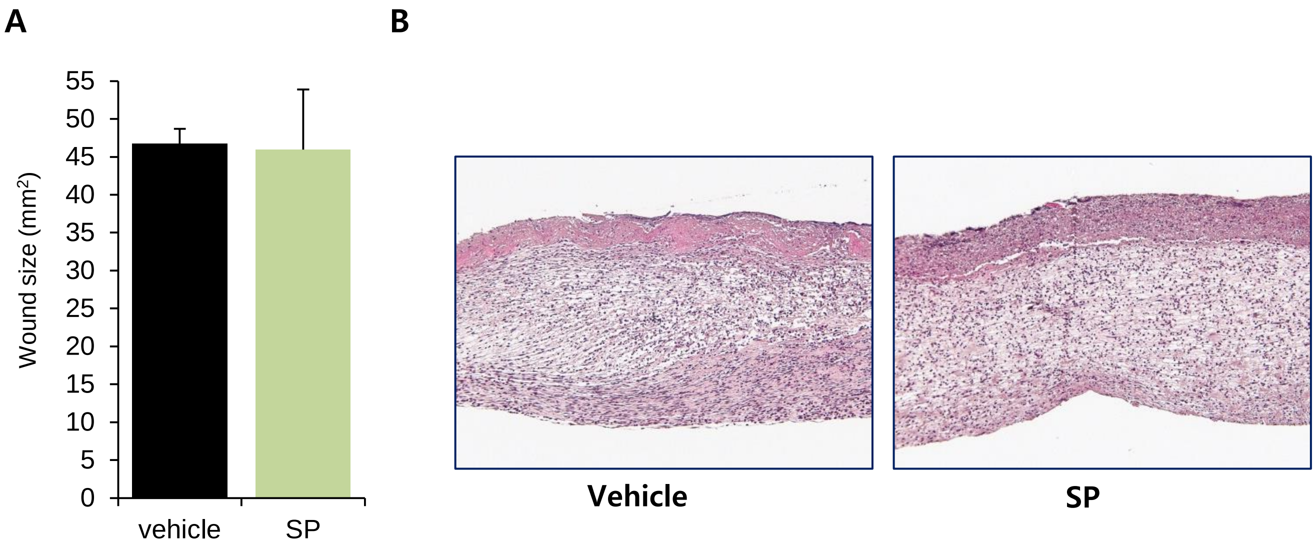


**Supplementary Figure 12.  The effect of SP on skin wound**

To check the effect of SP  on skin wound, SP was locally treated to wound site. 2 days later, wound size (A) and  skin tissue was analyzed by H&E staining (B)

# Supplementary table 1

| Passage no. | 3 | 8 |
|---|---|---|
| Marker | Percentage of expression (%) | |
| CD29 | 98. 2 ± 0.01 | 98.3 ± 1.5 |
| CD73 | 82.3 ± 0.09 | 79.4 ± 2.4 |
| CD105 | 94.2 ± 3.4 | 98.8 ± 1.5 |
| CD34 | 0.70 ± 0.01 | 0.8 ± 0.08 |
| CD90 | 89.7 ± 0.09 | 88.7 ± 0.4 |

**Supplementary table 1. Characterization of human MSC**

Human MSC (hMSC, 2×106, Passage number 3 and 8) were analyzed by flow cytometry analysis. For the detection of MSC surface makers, cells were treated with FITC-conjugated antibodies against CD29, CD73, CD105, CD34, and CD90 and analyzed with FACs.
